# Impact of molecular alterations on quality of life and prognostic understanding over time in patients with incurable lung cancer: a multicenter, longitudinal, prospective cohort study

**DOI:** 10.1007/s00520-021-06736-2

**Published:** 2021-12-07

**Authors:** Jonas Kuon, Miriam Blasi, Laura Unsöld, Jeannette Vogt, Anja Mehnert, Bernd Alt-Epping, Birgitt van Oorschot, Jochen Sistermanns, Miriam Ahlborn, Ulrike Ritterbusch, Susanne Stevens, Christoph Kahl, Anne Ruellan, Kathrin Matthias, Thomas Kubin, Kerstin Stahlhut, Andrea Heider, Florian Lordick, Michael Thomas, on behalf of the Arbeitsgemeinschaft Palliativmedizin (APM) of the German Cancer Society (DKG)

**Affiliations:** 1 Department of Thoracic Oncology, Thoraxklinik at Heidelberg University Hospital, Translational Lung Research Center Heidelberg TLRC-H, Member of the German Center for Lung Research DZL, Heidelberg, Germany; 2 Department of Medicine-2 (Oncology, Gastroenterology, Pulmonology, and Infectious Diseases), and University Cancer Center Leipzig (UCCL), University of Leipzig Medical Center, HepatologyLeipzig, Germany; 3 Department of Medical Psychology and Medical Sociology, University Hospital Leipzig, Leipzig, Germany; 4 Department of Palliative Medicine, Heidelberg University Hospital, Heidelberg, Germany; 5 Interdisciplinary Department of Palliative Medicine, University Hospital Würzburg, Würzburg, Germany; 6 Department of Radiation Oncology, Kliniken Maria Hilf, Mönchengladbach, Germany; 7 Department of Oncology and Hematology, Klinikum Braunschweig, Braunschweig, Germany; 8 Westdeutsches Tumorzentrum, University Hospital Essen, Essen, Germany; 9 Department of Internistic Oncology, Kliniken Essen Mitte, Essen, Germany; 10 Department of Hematology, , Oncology and Palliative Care, Klinikum Magdeburg, Magdeburg, Germany; 11 Department of Oncology, Hematology and Palliative Care, Städtisches Klinikum Karlsruhe, Karlsruhe, Germany; 12 Department of Hematology, Hemostasis, Oncology and Stem Cell Transplantation, Hannover Medical School, Hannover, Germany; 13 Department of Haematology Oncology and Palliative Care, Klinikum Traunstein, Traunstein, Germany; 14 Ambulatory of Haematology Oncology and Palliative Care, Immanuel Klinik Und Poliklinik Rüdersdorf, Rüdersdorf bei Berlin, Germany; 15 Department of Medicine 3, Klinikum Leverkusen, Leverkusen, Germany; 16 Department of Medicine 2 (Oncology, Gastroenterology, Hepatology, Pulmonology, and Infectious Disease), University Cancer Center Leipzig (UCCL), University of Leipzig Medical Center, Leipzig, Germany

**Keywords:** Lung cancer, Quality of life, Molecular alterations, Prognostic awareness

## Abstract

**Purpose:**

The purpose of this study is to investigate changes over time in quality of life (QoL) in incurable lung cancer patients and the impact of determinants like molecular alterations (MA).

**Methods:**

In a prospective, longitudinal, multicentric study, we assessed QoL, symptom burden, psychological distress, unmet needs, and prognostic understanding of patients diagnosed with incurable lung cancer at the time of the diagnosis (T0) and after 3 (T1), 6 (T2) and 12 months (T3) using validated questionnaires like FACT-L, National Comprehensive Cancer Network (NCCN) Distress Thermometer (DT), PHQ-4, SCNS-SF-34, and SEIQoL.

**Results:**

Two hundred seventeen patients were enrolled, 22 (10%) with reported MA. QoL scores improved over time, with a significant trend for DT, PHQ-4, and SCNS-SF-34. Significant determinants for stable or improving scores over time were survival > 6 months, performance status at the time of diagnosis, and presence of MA. Patients with MA showed better QoL scores (FACT-L at T1 104.4 vs 86.3; at T2 107.5 vs 90.0; at T3 100.9 vs 92.8) and lower psychological distress (NCCN DT at T1 3.3 vs 5; at T2 2.7 vs 4.5; at T3 3.7 vs 4.5; PHQ-4 at T1 2.3 vs 4.1; at T2 1.7 vs 3.6; at T3 2.2 vs 3.6), but also a worsening of the scores at 1 year and a higher percentage of inaccurate prognostic understanding (27 vs 17%) compared to patients without MA.

**Conclusion:**

Patients with tumors harboring MA are at risk of QoL deterioration during the course of the disease. Physicians should adapt their communication strategies in order to maintain or improve QoL.

## Introduction

Lung cancer is the most commonly diagnosed cancer and the leading cause of cancer-related death worldwide [1]. Despite significant advances in treatment, the prognosis remains poor, and the symptom burden seems to be higher in patients with advanced lung cancer compared to other tumors in advanced stage [2]. Moreover, patients with lung cancer have been shown to experience reduced quality of life (QoL) and emotional functioning [3], besides severe distress and a high number of unmet needs [2, 3].

In incurable cancer patients, QoL is determined by the prognosis and the treatments of the underlying disease, as well as by psychological distress such as anxiety and depression [6] and by sociodemographic characteristics [7]. Furthermore, QoL has been delineated as a prognostic factor for survival [8]. Previous studies have explored determinants of QoL in patients with advanced cancer using different validated questionnaires. They have identified older age, good Eastern Cooperative Oncology Group (ECOG) performance status (PS) and survival longer than 6 months as strong factors associated with better QoL [7, 9, 10]. Older patients and men have shown better physical and emotional functioning compared to younger patients and women, respectively [7, 9].

Additionally, adequate prognostic awareness and understanding represent the basic prerequisite for an appropriate end-of-life care [11]. An inaccurate comprehension of the prognosis and of goals of cancer treatment could lead to inadequate choices and administration of futile therapies at the end of life [12]. However, the effects of adequate comprehension of the prognosis on psychological distress and QoL remain unclear. Accurate prognostic awareness has been found to facilitate end-of-life-planning and appears to improve [13, 14] as well as to deteriorate [15, 16] QoL.

Certain molecular alterations in non-small cell lung cancer (NSCLC) have been increasingly described in the last decade as explanatory factors of the different clinical courses of the disease due to their role as therapeutic targets [17]. For example, epidermal growth factor receptor (EGFR) mutations, detected in approximately 10–20% of Caucasian and at least 50% of Asian NSCLC patients [18], or anaplastic lymphoma kinase (ALK) gene rearrangements, constituting about 4–5% of all NSCLC cases [19], represent good predictive factors because they can confer responsiveness to tyrosine kinase inhibitors (TKIs).

TKIs have demonstrated a significant survival advantage as compared to standard chemotherapy and a positive impact on symptoms [18]. Therefore, they can improve QoL in patients with advanced lung cancer compared to standard chemotherapy, as many studies have demonstrated [10, 11]. Moreover, these agents can be administered orally, and they tend to have more tolerable adverse events compared to chemotherapeutic agents, making them an attractive palliative treatment strategy [21].

However, the differences in QoL and prognostic awareness over time in lung cancer patients with or without detected molecular alterations have not been investigated.

The objective of the presented study was to assess QoL, symptom burden, distress, mood, and unmet needs of patients diagnosed with lung cancer from the diagnosis of incurability over time and to define possible correlations with patients’ characteristics.

## Methods

### Study design and sample

Our multicenter, prospective, longitudinal, observational study enrolled patients with advanced cancer to evaluate symptom burden, QoL, and associated factors during the course of advanced cancer treatments. The study concept was developed by task force members of the Palliative Medicine working group (APM) within the German Cancer Society (Deutsche Krebsgesellschaft, DKG). The study was funded by the DKG and approved by the local ethics authorities of all participating sites.

In the present analysis, we used data from patients diagnosed with incurable lung cancer who participated in our prospective study, expanding our first timepoint evaluation [20] with further assessments at 3, 6, and 12 months.

The recruitment took place in 12 German clinics with specialized departments in cancer care and oncology from 12/2014 until 10/2016. Patients with NSCLC at the diagnosis of incurability and before the start of any anticancer treatment were included. Further inclusion criteria comprised written informed consent and age > 18 years. Patients who were not able to understand and answer questions were not selected. Researchers approached individuals who met the eligibility criteria during a regularly scheduled clinical visit. After providing oral as well as written information and obtaining written informed consent, demographic and clinical data were recorded in case report forms, as well as details about therapeutic choices. The presence of molecular alterations (EGFR mutations and ALK translocation) was assessed and recorded whenever this information was available.

### Measures

Multiple self-assessment instruments were administered in order to characterize comprehensively symptom burden (Functional Assessment of Chronic Illness Therapy-Lung, FACT-L), distress (National Comprehensive Cancer Network (NCCN) distress thermometer), depression and anxiety (Patient Health Questionnaire, PHQ-4), unmet needs (Supportive Care Needs Survey, SCNS-SF-34-G modified), and quality of life (Schedule for the Evaluation of Individual Quality of Life, SEIQoL). Patients completed measures of quality of life at different timepoints: at the time of diagnosis of the incurability (T0) and after 3 (T1), 6 (T2), and 12 months (T3). We also collected information about their understanding of treatment goals (cure, prolonged life, or maintained/improved quality of life). Prognostic awareness was investigated by asking the patients about the treatment goal: patients that indicated “cure” as the treatment goal were considered as having an inaccurate prognostic awareness.

#### FACT-L

The FACT-L scale is a 36-item instrument for the measurement of multidimensional quality of life and is a combination of the 27-item FACT-General (FACT-G) and the 9-item Lung Cancer Subscale (LCS). The FACT-G score is obtained from the sum of the physical well-being (PWB), social/family well-being (SWB), emotional well-being (EWB), and functional well-being (FWB) subscales scores. The score range from 0 (worst) to 136 (best quality of life) [21].

#### NCCN distress thermometer

The NCCN distress thermometer is a single-item visual analogue scale ranging from 0 (no distress) to 10 (extreme distress) on which patients are asked to rate their overall distress in the last week [22].

#### PHQ-4

PHQ-4 is a two-item ultra-brief screening tool for depression and anxiety. It is obtained from the sum of PHQ-2, which evaluates criteria for depression, depressive mood, and anhedonia, with GAD-2 (Generalized Anxiety Disorder Scale-2), a 2-item screening for generalized anxiety. The total PHQ-2 and GAD-2 score ranges from 0 to 6, and the composite PHQ-4 total score ranges from 0 to 12 [23]. Scores are rated as normal (0–2), mild (3–5), moderate (6–8), or severe (9–12) depression and anxiety.

#### SCNS-SF-34-G modified

The modified SCNS-SF-34-G is a tool for the assessment of supportive care needs. This modified version is composed from 25 items instead of 34. They address psychological needs, health system and information needs, physical and daily living needs, patient care and support needs, and sexuality needs. For each item, patients were asked to indicate their level of need related to cancer during the last month. Each item was scaled from 1 (“not applicable”) to 5 (“high need”). A score > 2 indicates that a particular item is an unmet need [24]. For our analysis, we considered the number of unmet needs for each patient.

#### SEIQoL

SEIQoL is a self-reporting tool for the evaluation of the importance of 12 specific life domains and their respective satisfaction. An individual QoL index can be calculated, and it can range from 0 to 100, with higher values representing a better QoL [25].

### Statistical analyses

Data were first descriptively analyzed to estimate frequencies, means, and standard deviation of the variables. We used univariate mixed-effect linear model for repeated measures to assess changes over time in the scores and factors that significantly influenced the scores’ trends over time accounting for missing values. The univariate analysis was performed for the following independent factors: age group (< 65 and ≥ 65), gender, survival (< 6 months and ≥ 6 months), performance status (PS) Eastern Cooperative Oncology Group (ECOG), and presence of detected molecular alterations such as EGFR mutations or ALK translocation. All scores are presented as means (standard deviations). *P* values < 0.05 were considered significant. We conducted statistical analysis using SPSS v. 25.0 for Windows (SPSS Inc., Chicago, IL).

## Results

### Patients’ characteristics

Clinical and demographic baseline characteristics are provided in Table 1.

**Table 1:** Patient characteristics at the baseline (*n* = 217)

Characteristic		*N* (%)
Age	< 65 ≥ 65	119 (55) 98 (45)
Gender	Male	128 (59)
	Female	89 (41)
Molecular alteration	EGFR mutation	17 (8)
	ALK translocation	5 (2)
Smoking history	yes no	180 (82) 37 (18)
ECOG PS	0	46 (21)
	1	110 (51)
	2	46 (21)
	3	14 (7)
	4	1 (0)
Tumor stage	Locally advanced	34 (16)
	Metastatic	183 (84)

We evaluated 217 patients. The mean age was 63.6 years (25–86); 128 patients were male (59%).

Twenty-one percent were classified as having a baseline PS ECOG 0, 51% as grade 1, 21% as grade 2, and 7% as grade 3. In 22 patients (10%), molecular alterations (EGFR mutations or ALK translocation) were detected.

Twenty of 22 patients received frontline TKIs (erlotinib, afatinib, or gefitinib in case of EGFR mutation and crizotinib or ceritinib in case of ALK translocation), one patient received first-line chemotherapy, and one patient received best supportive care only.

The sample size decreased at each assessment time point because of patients’ death or missing data due to drop out or patients lost to follow-up. Detailed numbers are provided in Table 2. After 1 year, 78 patients were alive. In detail, 17 of 22 (77%) patients with reported molecular alteration were alive at T3.

**Table 2:** Changes in sample size over time

	T1 ***N* (%)**	T2 ***N* (%)**	T3 ***N* (%)**
Alive	160 (74)	119 (55)	78 (36)
Dead	46 (21)	87 (40)	128 (59)
Missing	11 (5)	11 (5)	11 (5)

### QoL scores over time

QoL scores showed a progressive improvement during the first 6 months and psychological distress decreased over time. Means and standard deviations for each questionnaire’s score are reported in Table 3.

**Table 3:** QoL scores over time

	**T0** (*n* = 217)	**T1** (*n* = 160)	**T2** (*n* = 116)	**T3** (*n* = 78)
FACT-L	88.0 (20.8)	88.5 (21.7)	92.9 (21.9)	93.8 (21.0)
PHQ-4	4.6 (3.3)	3.9 (3.0)	3.3 (2.7)	3.3 (3.0)
SEIQoL	60.8 (12.2)	61.1 (13.4)	63.0 (12.0)	63.8 (11.7)
NCCN DT	5.4 (2.7)	4.8 (2.6)	4.3 (2.6)	4.4 (2.8)
SCNS	9.1 (8.7)	6.7 (7.7)	6.3 (7.8)	5.0 (6.8)

The mixed effect linear model for repeated measures analysis showed a statistically significant trend over time for NCCN DT, PHQ-4, and SCNS questionnaires (Table 4).

**Table 4:** Changes in scores trend and factors influencing the trend over time for FACT-L, NCCN DT, PHQ-4, SCNS and SEIQoL (A) and FACT-L subscales (B). *P* values for any single univariate model

	**A**					**B**				
	FACT-L	NCCN DT	PHQ-4	SCNS	SEIQoL	PWB	SWB	EWB	FWB	LCS
Trend over time	0.945	0.024	0.028	< 0.001	0.320	0.308	0.439	0.014	0.853	0.314
Age group	0.995	0.622	0.145	0.006	0.313	0.749	0.225	0.259	0.417	0.053
Gender	0.539	0.356	0.324	0.804	0.926	0.541	0.740	0.412	0.569	0.837
Alive at T2	0.012	0.003	< 0.001	0.336	< 0.001	0.001	0.599	0.031	< 0.001	0.012
PS ECOG	< 0.001	< 0.001	< 0.001	0.284	< 0.001	< 0.001	0.017	< 0.001	< 0.001	< 0.001
Molecular alterations	0.003	0.045	0.048	0.620	0.287	0.002	0.419	0.108	0.005	0.007

Survival longer than 6 months after the diagnosis and PS ECOG were significantly associated with changes in scores’ trend over time. Patients aged 65 years or older did not show remarkable differences between the scores compared to younger patients, except for SCNS-SF-34-G. Younger patients reported more unmet supportive care needs compared to the older ones at each timepoint: 10.3 vs 7.6 at T0, 7.7 vs 5.5 at T1, 7.3 vs 5.1 at T2, and 5.7 vs 3.9 at T3, respectively.

Although gender was not identified as a significant determinant for the score trends over time, female patients reported higher levels of distress at the diagnosis compared to male patients (NCCN DT 5.9 vs 5.0). After 3 months, the mean DT score was 4.8 for both gender types. At 6 and 12 months, however, women reported lower distress levels compared to men (3.6 vs 4.8 at T2 and 3.9 vs 4.6 at T3). The same trend was noted for the PHQ-4 questionnaire, with women reporting more anxiety and depression at the baseline and this trend reversing at 6 months. PHQ-4 scores were 5.1 vs 4.2 at T0, 4.0 vs 3.8 at T1, 3.0 vs 3.5 at T2, and 2.8 vs 3.6 at T3 for women and men, respectively. Moreover, FACT-L scores were comparable between the two groups at T0 and T1, while women reported better scores at the last two times: 98.1 vs 88.9 at T2 and 98.4 vs 91.7 at T3.

**Fig. 1:**
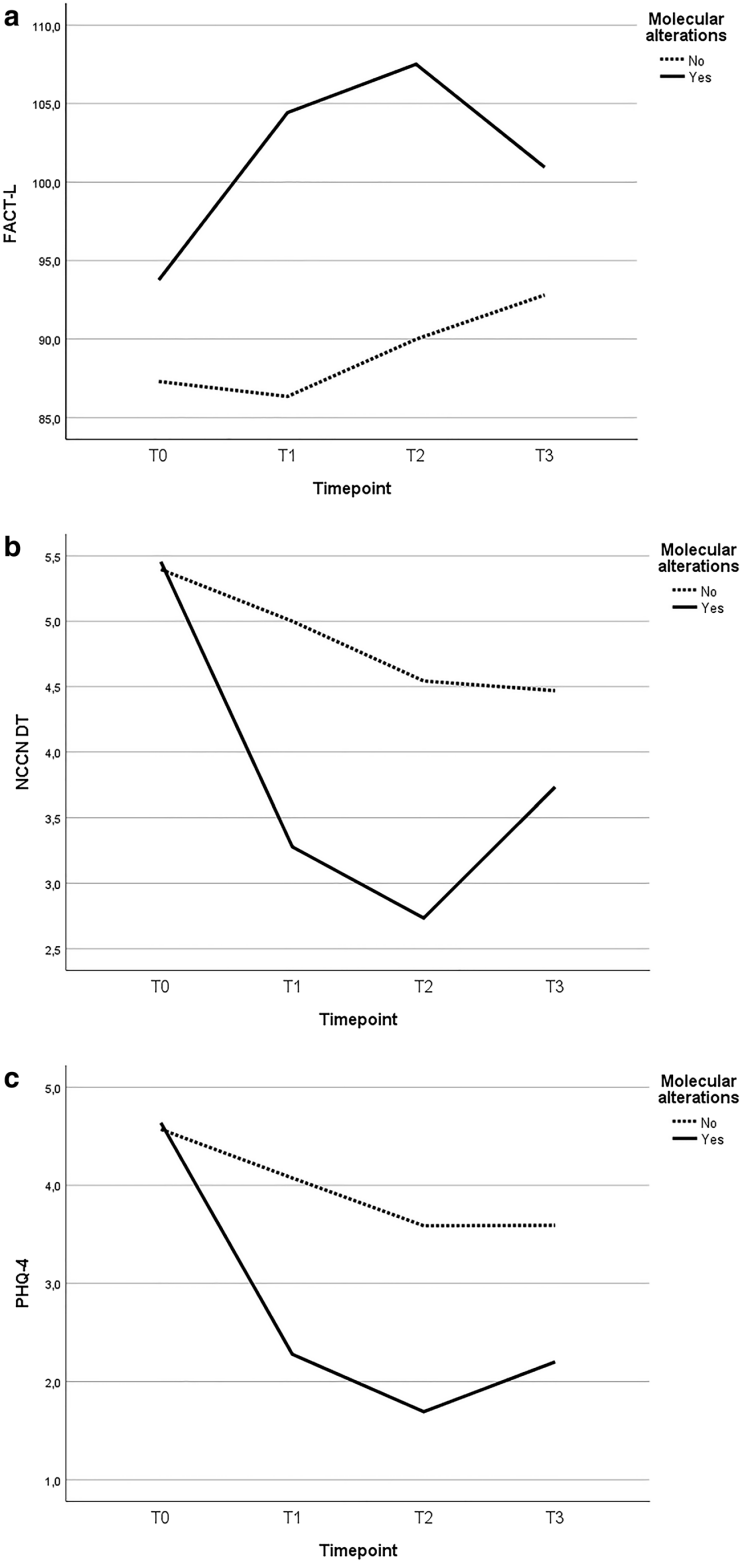
**A** FACT-L (higher scores indicate better QoL), **B** NCCN DT (lower scores indicate less distress), and **C** PHQ-4 (lower scores indicate less psychological impairment) trends over time in patients with (yes) or without (no) detected molecular alterations

Half of the patients with reported EGFR mutations or ALK translocation were male, and nearly 40% were never smoker. The mixed effects linear model for repeated measures analysis showed that the presence of molecular alterations influenced FACT-L, NCCN DT, and PHQ-4 scores’ trend. Between the FACT-L subscales, PWB, FWB, and LCS were also influenced by the molecular status. Compared to patients without reported molecular alterations, patients with EGFR mutations or ALK translocation showed higher FACT-L scores over time (at T0 93.8 vs 87.3; at T1 104.4 vs 86.3; at T2 107.5 vs 90.0; at T3 100.9 vs 92.8). NCCN DT and PHQ-4 scores were comparable at baseline between the two groups, but they showed a favorable trend for patients with molecular alterations over time (NCCN DT at T1 3.3 vs 5; at T2 2.7 vs 4.5; at T3 3.7 vs 4.5; PHQ-4 at T1 2.3 vs 4.1; at T2 1.7 vs 3.6; at T3 2.2 vs 3.6). No significant difference was noted regarding SCNS-SF-34-G and SEIQoL scores between the two groups. Moreover, in patients with molecular alterations, a worsening of the scores at T3 compared to T2 was noted, and this trend was not evident in patients without molecular alterations. Scores’ trend for the two groups is shown in Fig. 1.

### Prognostic awareness over time

The proportion of patients with inaccurate prognostic awareness, that means to consider “cure” as treatment goal, decreased over time, from 29% at T0 to 19% after 1 year.

A higher percentage of patients with detectable molecular alterations had an inaccurate prognostic understanding of the prognosis compared to those without molecular alterations at the time of the diagnosis (36% vs 29%, respectively) and after 1 year (27% vs 17%, respectively).

## Discussion

In this prospective longitudinal observational study, we assessed QoL encompassing symptom burden, psychosocial aspects, and prognostic understanding of incurability in patients newly diagnosed with incurable lung cancer.

With a median age of 63 years, the patients in this survey represent a younger cohort compared to real-world populations of incurable lung cancer patients. Lung cancer is predominantly a disease of the elderly, with a median age at the diagnosis of 70 years and almost 70% of patients diagnosed after 65 years of age [26]. However, older patients are under-represented in clinical trials with only 25% of them historically enrolling patients older than 65 years [27], yielding serious difficulties evaluating efficacy and safety of the different treatment options. Here it is evident, that even in a non-interventional observational survey the participants are younger than in the real-world settings. This finding could be explained by the fact that multiquestionnaire self-assessment are often not feasible for older patients, as well as that older patients with multiple comorbidities and poorer PS could be more inclined to refuse enrollment in a clinical study.

The drop-out rate at each timepoint, mostly due to death, was 26.3% at T1, 45.2% at T2 and 64.1% at T3, respectively. After 1 year, 35.9% of the patients were alive.

Dropout in longitudinal trials is common, especially in advanced lung cancer populations, and a potential source of bias. Indeed, patients with poor baseline QoL are usually those with a poor prognosis; thus, they have a worse disease trajectory and a faster QoL deterioration. These patients commonly drop out earlier than those with better baseline QoL [28]. Accordingly, survival time > 6 months was recognized as a significant determinant for QoL, as previously described by others [9], and this was valid for almost every questionnaire and subscale. For these reasons, multiple missing data are unavoidable in longitudinal quality of life datasets in the advanced NSCLC population, leading to a selection bias and affecting results and conclusions. Accordingly, missing data in repeated measurements over time requires statistical analysis techniques capable of dealing with these issues, such as univariate mixed-effect linear model for repeated measures [30].

Measured questionnaires’ scores remained stable or slightly improved over time, and this was consistent among all questionnaires.

These findings could additionally been explained by the development of coping mechanisms and response shift, a change of standards in which the patient may judge the level of fatigue differently from how he would have judged it before to experience extreme fatigue [29].

Patients aged 65 years or older showed significant differences in SCNS-SF-34-G questionnaire compared to younger patients, with the latter reporting more unmet supportive care needs over time. Several studies have investigated differences in QoL between young and old cancer patients. Many of these studies have revealed that older subjects have better emotional and social functioning and worse physical functioning compared to those of their younger counterparts, but looking at their global QoL, no significant differences were found [30–32]. This was confirmed by our study, in which only differences in unmet needs were documented. Watson et al. showed only few differences in unmet needs between senior (≥ 65 years) and junior (< 65 years) cancer patients, except for psychological and sexuality domains [33]. This correlates with the previous report from Akechi et al. [32]. Our results are in accord with these previous reports [32, 33], showing a decrease of patients’ unmet needs over time.

Women reported higher distress levels and more psychological impairment compared to men at T0, as previously reported [34]. However, women showed an improvement in these aspects over time, while men did not. This observation could be explained by the fact that women use psychological services more frequently than men do [35], while male patients are less likely to reveal their emotional distress [36].

Targetable molecular alterations such as EGFR mutations and ALK translocation were reported in 10% of our patients. These patients showed less depression (PHQ-4) and distress (NCCN DT) and higher FACT-L scores along the observation time, although the baseline scores were comparable between the two groups. The 77% of patients from this group were alive at 1 year, compared to the 36% of the entire cohort.

Several observational studies have shown that EGFR-mutant NSCLC is associated with lower depression rates and severity [37, 38]. A positive correlation between depression and inflammation in multiple cancer settings, including lung cancer [39, 40], is postulated. In a recent publication, less depression in EGFR mutant NSCLC patients was documented, probably mediated by lower CRP‐related inflammation [17]. It is a new question, whether the relationship between EGFR mutational status and depression is mediated by other mechanisms, besides inflammation, that protect patients from psychological impairment.

It is known that EGFR-mutant NSCLC is generally associated with a more indolent disease course since over 85% EGFR mutant NSCLC respond to TKIs, even if it is only for a prescribed and limited period of time [41]. Of the 22 patients with reported molecular alterations included in our analysis, 20 received TKIs as frontline treatment.

Interestingly, patients with molecular alterations showed psychological deterioration at 12 months, with increased distress and depression levels and worse FACT-L scores. This could be explained by the fact that patients usually respond to EGFR inhibitors for 9–13 months before showing disease progression [42]. As recently published in a cohort study enrolling more than 2000 cancer patients with breast, pancreatic, lung, and colon cancer, disease progression is associated with a deterioration in global health-related QoL that was markedly higher in lung cancer patients [43].

Our group of patients with molecular alterations also comprises ALK-positive patients, a distinct molecular subtype of NSCLC. In these patients, sequential administration of ALK TKIs results in a median overall survival exceeding 5 years [44] with clinically meaningful improvement in QoL [45].

Although QoL scores were better at each time point in patients with molecular alterations, NCCN DT and PHQ-4 were comparable between the two groups at the baseline.

To our knowledge, this is the first report comparing distress level and grade of depression and anxiety prior to treatment initiation and over time in lung cancer patients with and without detectable targetable mutations.

At baseline, 29% of patients reported an inaccurate prognostic awareness, and this percentage decreased over time. Limited data are available on development and changes in prognostic awareness over time. The link between prognostic awareness, QoL, and psychological distress remains unclear, with discordant and controversial results available in literature [11]. Interestingly, patients with targetable driver mutations reported more frequently an inadequate prognostic understanding. This could also be an explanation for the worsening of FACT-L, NCCN DT, and PHQ-4 scores after 1 year: when molecular alterations are detected, a better prognosis is envisaged to patients, despite the development of resistance to TKIs is an unavoidable process. This is, to our knowledge, the first time this phenomenon has been described in a longitudinal trial.

Using a comprehensive assessment evaluating different dimensions of QoL, we underlined the dynamic changes of distinct domains and the factors that may influence these changes. Periodical QoL assessments are time-consuming and not applicable to real-life practice. In the era of targeted treatment, future research should aim at investigating how QoL and psychological distress evolve during the disease course of patients treated with novel agents, thus how treating physicians can approach these patients in order to provide a personalized support strategy with appropriate timing.

## Limitations

Our study has some limitations. First, the median age in our cohort was younger than expected in a non-selected cohort of incurable lung cancer patients. For this reason, our findings should be transfer with caution into real-world settings. Second, no information about the time of progression was provided. Third, it is possible that participants with severely impaired health status and PS have not been included in this observation, resulting in a younger cohort than expected, an underestimation of QoL deterioration and symptom burden. In addition, information about screening failure and patients that refused the enrollment was not available.

However, the strength of the study is its prospective design and the longitudinal assessment of multiple quality of life domains compared to most of the other published studies.

## Conclusion

In the era of precision oncology, personalized approaches to improve QoL should be implemented. This customization should comprise sociodemographic factors but also predictive biological markers. This is particularly important in a very heterogeneous disease such as lung cancer, in which biological characteristics allow the identification of subtypes with different prognosis and predict efficacy of new treatment strategies. Further research should focus on the impact of these factors on QoL to help physicians to guide patients through the disease trajectory.

## Data Availability

Available from the authors on request.
